# Diffusive micromixing combined with dynamic in situ laser scattering allows shedding light on lipid nanoparticle precipitation

**DOI:** 10.1038/s41598-024-73721-0

**Published:** 2024-10-17

**Authors:** Ebrahim Taiedinejad, Cornelius Bausch, Jörn Wittek, Gökhan Gül, Peer Erfle, Nicolai Schwarz, Mohadeseh Mozafari, Michael Baßler, Andreas Dietzel

**Affiliations:** 1 Technische Universität Braunschweig, Institut für Mikrotechnik, Alte Salzdahlumer Str. 203, 38124 Braunschweig, Germany; 2 Zentrum für Pharmaverfahrenstechnik (PVZ), Technischen Universität Braunschweig, Franz-Liszt-Str. 35a, 38106 Braunschweig, Germany; 3 Fraunhofer-Institut für Mikrotechnik und Mikrosysteme IMM, Carl-Zeiss-Str. 18-20, 55129 Mainz, Germany

**Keywords:** Microfluidics, Two-photon-polymerization, Lipid nanoparticle precipitation, Dynamic light scattering, In-situ measurement, Engineering, Nanoscience and technology, Optics and photonics

## Abstract

Pharmaceutical formulations are increasingly based on drug nanoparticles or carrier nanoparticles encapsulating drugs or mRNA molecules. Sizes and monodispersity of the nanoparticles regulate bioavailability, pharmacokinetics and pharmacology. Microfluidic mixers promise unique conditions for their continuous preparation. A novel microfluidic antisolvent precipitation device was realized by two-photon-polymerization with a mixing channel in which the organic phase formed a sheet with a homogeneous thickness of down to 7 μm completely wrapped in the aqueous phase. Homogeneous diffusion through the sheet accelerates mixing. Optical access was implemented to allow in-situ dynamic light scattering. By centering the thin sheet in the microchannel cross-section, two important requirements are met. On the one hand, the organic phase never reaches the channel walls, avoiding fouling and unstable flow conditions. On the other hand, in the sheet positioned at the maximum of the parabolic flow profile the nanoparticle velocities are homogenized which enables flow-compensated Dynamic Light Scattering (*flowDLS*). These unique features allowed in-situ particle size determination for the first time. Monitoring of lipid nanoparticle precipitation was demonstrated for different rates of solvent and antisolvent flows. This breakthrough innovation will not only enable feedback control of nanoparticle production but also will provide new insights into the dynamics of nanoparticle precipitation.

**Supplementary Information:**

The online version contains supplementary material available at 10.1038/s41598-024-73721-0.

## Introduction

Nanoparticles are finding a huge market in today’s world in the fields of pharmaceuticals, food and cosmetics^1–3^. A number of physical properties influence their in-vivo performance, particularly safety, stability and efficacy^4^. Lipid nanoparticles are being used for drug delivery due to their biocompatibility, ability to protect the encapsulated drug, and controlled release properties. They also offer surface functionalization for precise distribution to specific cells and tissues. Lipid nanoparticles have shown promise in cancer and infectious disease treatment, with recent mRNA vaccines showing effectiveness in treating serious illnesses like SARS-CoV-2^5^. The effectiveness of nanoparticles for drug and RNA delivery depends on their ability to carry formulations and maintain a stable concentration until they reach their targets. Applications of polymer nanoparticles are limited due to cost and, in some cases, safety issues^6–8^. Lipid-based nanoparticles (*LNPs*) have been developed as an alternative to overcome these limitations, making them one of the most promising techniques for delivering drugs^9–11^. They enable a variety of administration routes such as oral, dermal, pulmonary, ophthalmic and buccal administration^2,12,13^.

Solubility is one of the important parameters to achieve the desired concentration of a drug in systemic circulation for the desired pharmacological response^8^. Around 40% of molecules fail in the drug development process due to poor solubility and bioavailability and the production of these particles in sufficiently high pharmaceutical quality still poses a challenge^14–17^. Various studies have shown that the size of the drug delivery systems influences pharmacokinetics and tissue response, distribution and clearance^18–23^. The quality of a formulation depends largely on the average particle size and the size variation measured as the polydispersity index (PDI)^24^. Several studies on the pharmacokinetic behaviour of intravenously injected nanoparticles have shown that particles between 70 and 100 nm and up to 200 nm are most suitable, particularly for cancer treatment^12,25–27^.

Nanoparticles are typically produced by top-down mechanical methods such as high-pressure homogenization, wet grinding, or ultrasonication of coarser particles that have already been prepared^17,28,29^. However, high temperatures, high shear stress, high pressure or product contamination due to mill erosion can cause damage to the active ingredients. Another option of lipid nanoparticle production as carrier systems or nanoparticulate drugs appears with bottom-up approaches^17^. These low-energy processes enable the generation of particles from molecular drug solutions and result in particle sizes of less than 100 nm and narrow size distributions. For precipitation methods, the active substance or the carrier system, e.g., a lipid, is dissolved. In a subsequent step, the solubility of the lipid in the solution is greatly reduced and supersaturating of the solution occurs, causing the dissolved substances to precipitate in the form of nanoparticles. Such precipitation can take place by solvent injection and solvent diffusion. This leads to a lower mechanical and thermal load on the nanoparticles, which is a crucial prerequisite for thermo-labile active ingredients^25,30–33^.

Microfluidic systems with typical channel dimensions of 500 μm and less offer the possibility to perform precipitation with much better control of mass and heat transfer in a continuous process^34–37^. Due to the small dimensions of the systems, reproducible and controlled processes for continuous production of nanoparticles can be realized^38^. It is crucial for the quality of the nanoparticles that the mixing proceeds as fast and evenly as possible so that the supersaturation is uniform throughout the system^39,40^.

In order to achieve small particle sizes, only primary nucleation should take place and the particles should have little time to continue growing after a critical nucleus has been created. To achieve this, the mixing should be faster than precipitation^41^. However, the production of these particles in sufficiently high pharmaceutical quality still poses a challenge^32,33,42–44^. Recently, a new generation of microfluidics was presented which allows continuous production of *LNPs* with unparalleled precision in the control of precipitation using mixers with complex 3D geometries made possible by two-photon-polymerization (*2PP*)^45–47^.

Typically, the quality of the produced particles is evaluated after completion of the production process by measuring the size distribution. This involves manual sampling followed by an offline analysis. The most commonly used method is dynamic light scattering (DLS), which can determine the Brownian motion of particles by analyzing the temporal evolution of laser speckles resulting from elastically scattered light^48^.

There have been efforts to apply *DLS* analysis to microfluidic channels where the typical Hagen-Poiseuille flow implies a parabolic velocity distribution^49–52^. Solutions so far work correctly only at very low flow rates and cease functioning at typical flow rates for microfluidic nanoparticle synthesis.

Recently, a measurement instrument was presented which employs spatially resolved *DLS* measurements using optical coherence tomography (DLS-OCT), thus enabling size characteristics measurements of flowing particles^53^. This instrument however can only measure near the channel wall, which makes it unsuitable for the direct measurement of particles synthesized in microfluidic devices since the precipitated particles are preferably located in distance to the channel walls to avoid fouling. Additionally, complex optical components are required for DLS-OCT^54,55^.

Here, we describe a novel microfluidic method, based on the previously developed *flowDLS* method^55^, that for the first time enabled in-situ particle analysis in a fast micromixer. This was made possible by the unique design of a low aspect ratio lamination mixer (LARLM) that was fabricated by *2PP* and allowed not only a controlled continuous LNP production, but also a nanoparticle flow with the required homogeneous velocity and without contact to the microchannel walls.

## Materials and methods

### Microfluidic chip fabrication

**Fig. 1:**
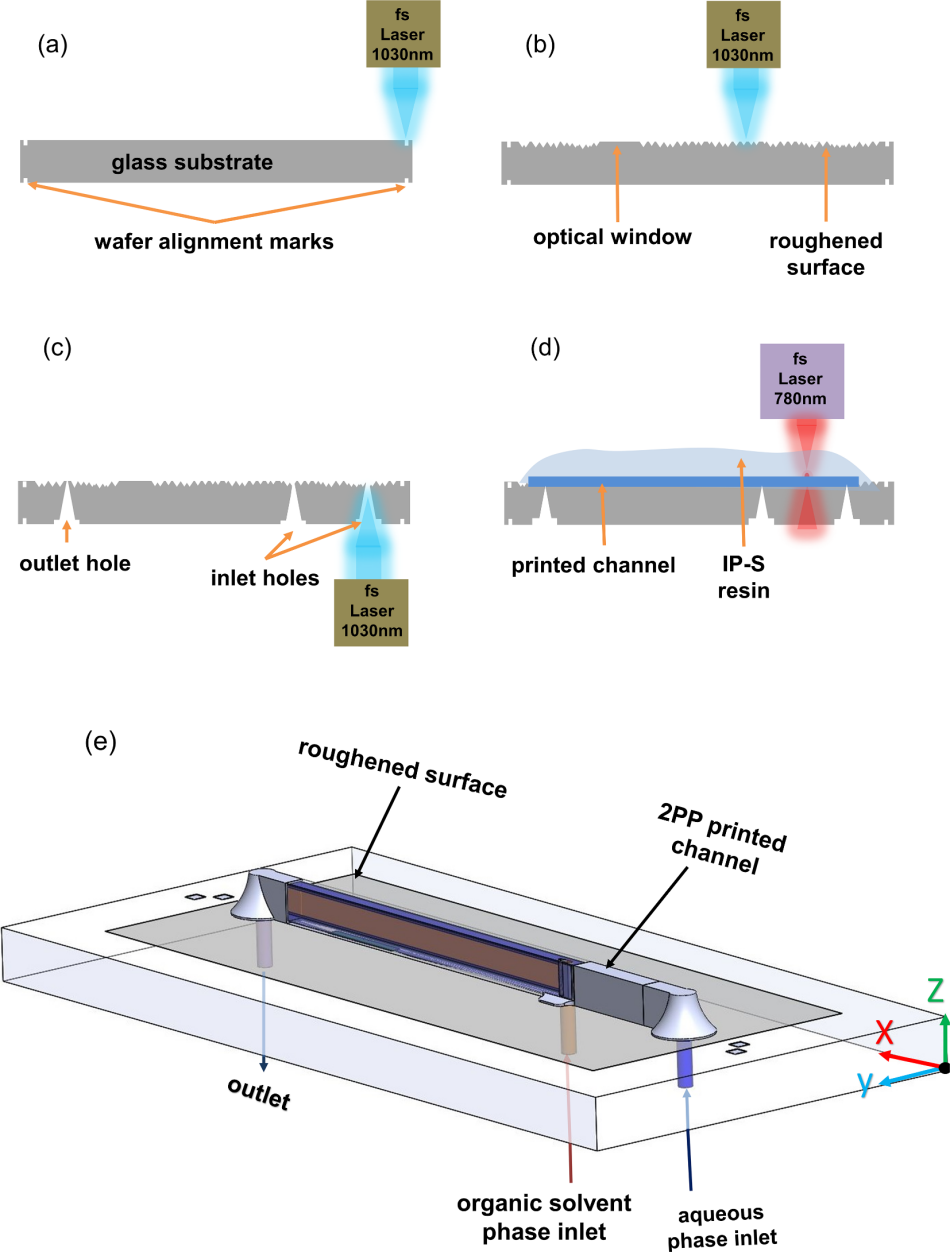
A schematic representation of the chip fabrication processes. By using fs laser ablation (**a**) alignment marks, (**b**) surface roughening excluding optical windows for the *DLS* laser beam and (**c**) outlet and inlet holes were created. (**d**) Microchannels were printed on individual chips onto the roughened glass areas in *2PP* photoresist. (**e**) A sketch of the LARLM chip with the rectangular main channel, which has a cross section of 360 × 750 µm^2^ and a length of 14.5 mm. Part of the microchannel between solvent phase inlet and outlet is shown transparent to view the injected organic solvent phase (brown color).

4-inch borosilicate glass wafers with a thickness of 1.1 mm (BOROFLOAT^®^ 33 from Schott, Mainz, Germany) exhibiting a high degree of flatness and excellent optical quality formed the base material of the microfluidic chip. A micro structuring system (microSTRUCT c, 3D Micromac AG, Chemnitz, Germany) equipped with a femtosecond laser (Pharos-10 W from Light Conversion Vilnius, Lithuania) emitting at its fundamental wavelength of 1030 nm with a pulse frequency of 100 kHz was used to locally roughen the surface of the glass, to create through holes, and to apply alignment marks for the generation of microchannels by *2PP*. The alignment marks were located on both sides of the wafer (see Fig. 1a). For each structure, the laser spot was moved by a galvanometer scanner (Scanlab RTC5, Puchheim, Germany) with a speed of 2000 mm s^− 1^ along parallel lines. After one full pass, this fill structure was rotated by 45°, and after every fourth pass, the laser focus was moved 50 μm deeper into the substrate. Pulse energies of 6.4 µJ and 22.6 µJ were used for roughening and through-hole drilling, respectively. These laser ablation steps are illustrated in Fig. 1a–c after which the wafer was diced into small chips of 19 × 9.5 mm^2^^44^.

The microchannel structures were printed on individual chips by means of a *2PP* system (Photonic Professional GT2, Nanoscribe GmbH, Eggenstein-Leopoldshafen, Germany) exposing voxels with sub-micron lateral dimensions in a light-curing resist (Nanoscribe’s IPS negative-tone photoresist with Young’s modulus of 5.1 GPa and reflection index of 1.515) as sketched in Fig. 1d. The laser emitted light pulses of less than 90 fs at 780 nm with a repetition rate of 80 MHz. The laser power was set to a value of 63 mW. The laser was focused by a microscope objective lens (LCI “Plan-Neofluar” 25×/0.8 Imm Korr Ph2, Zeiss) with 25× magnification and a numerical aperture of 0.8. For the writing, the objective lens was directly immersed in the photoresist. In z-direction, the object to be printed was divided into individual layers with a height of 1 μm. The objective lens provided a maximum working space of 285 × 285 × 300 µm^3^ (xyz directions); therefore, larger objects were split into blocks. With a galvanometer scanner, the laser spot was moved laterally over the substrate at a speed of 100 mm s^− 1^. For the vertical to the next layer, the substrate was positioned with the piezo stage. After the completion of a block the stage was moved to the next position. After laser exposure, the non-polymerized photoresist was removed in a mr-Dev 600 developer bath (Micro resist technology GmbH, Berlin, Germany). Resist residues still remaining in the microchannels were extracted by suction from the channel outlet, while the developer solution was pressed into one of the channel inlets. After subsequent drying, the printed object was cured on a hotplate at 190 °C for 10 min. The printed structures had high transparency and hydrophobic surface properties. Locally roughened glass ensured good adhesion of the microchannels.

### Microfluidic system design

**Fig. 2:**
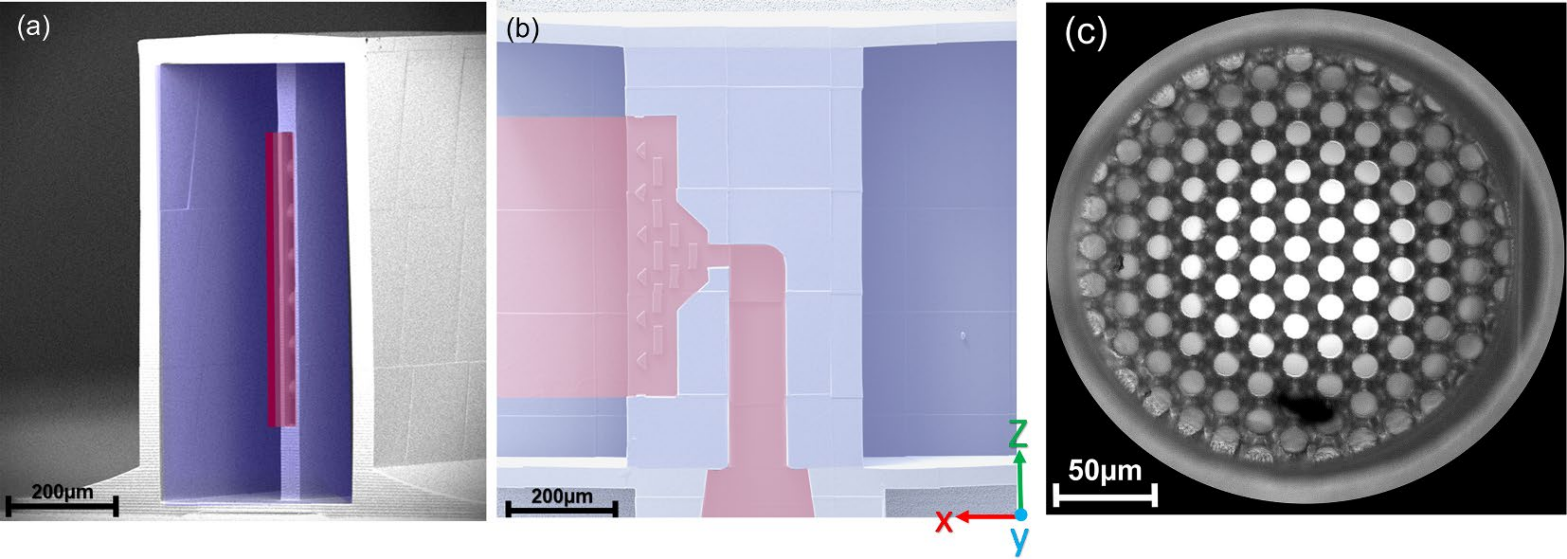
Scanning electron microscope (SEM) images of (**a**) the inside of the microchannel, which was printed incompletely to give an unobstructed view of the nozzle. (**b**) The nozzle interior (half of the nozzle was not printed with four rows of flow homogenisation barrier elements. Digital colouring indicates the liquid phases (red: organic, blue: aqueous). (**c**) Light microscopic image of an inlet showing the filter with a pore size of 7 μm in the center.

A drawing of the low aspect ratio lamination mixer (*LARLM*) chip with details of the microchannel is shown in Fig. 1e. In order to ensure a homogeneous injection of a thin sheet of organic solvent into the center of the main channel, a nozzle with an outlet cross-section of 20 ×  500 µm^2^ (aspect ratio of 1:25) was implemented, as shown in Fig. 2a. A homogeneous injection velocity was achieved by barrier elements within the nozzle, as shown in Fig. 2b. The *LARLM* was designed to include *2PP* printed filters with a pore diameter of 7 μm. The filters as shown in Fig. 2c were integrated at the channel inlets and led to a substantial improvement in the process stability and lifetime of the devices.

### Experimental setup

**Fig. 3:**
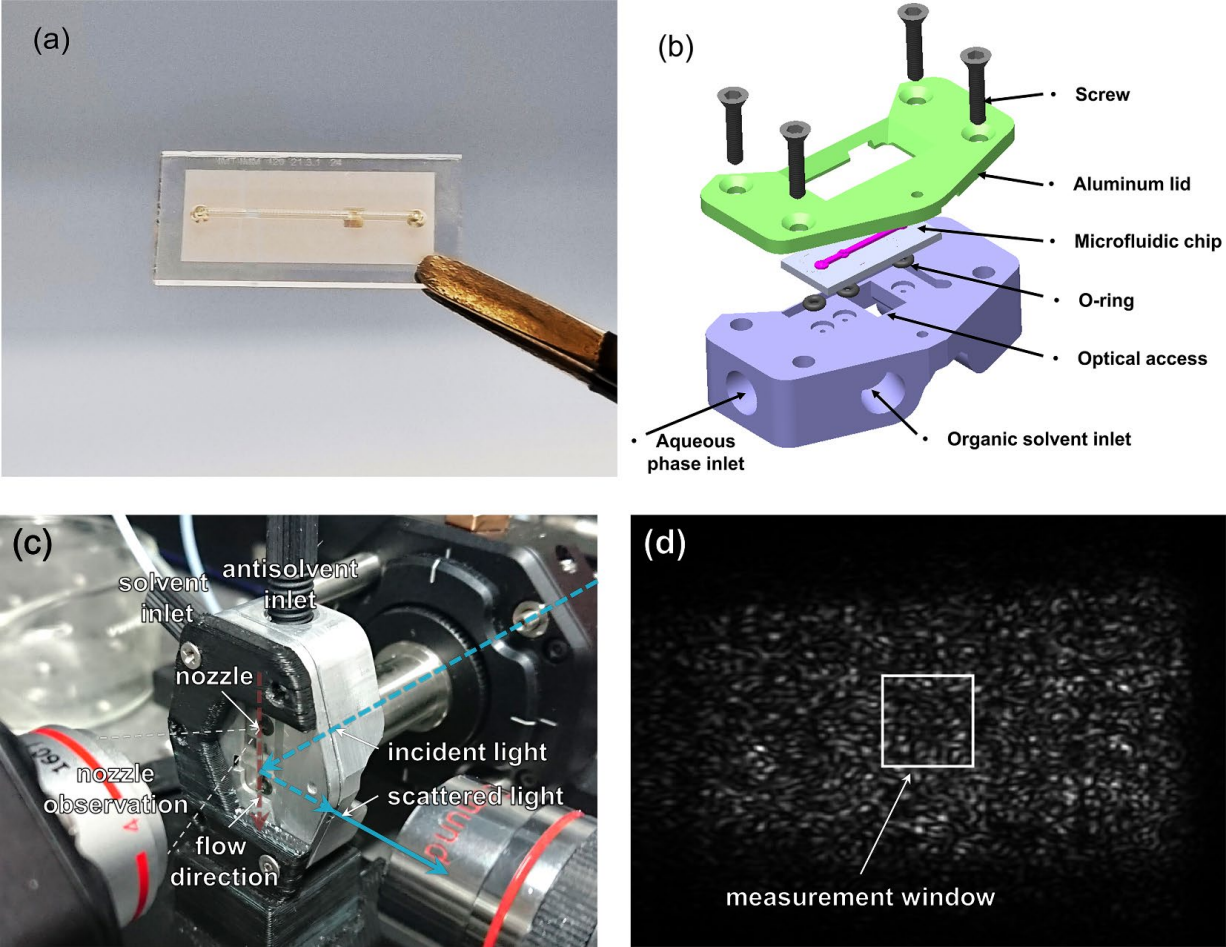
(**a**) Photograph of a finished LARLM chip. (**b**) Exploded view of the mounting bracket assembly with the chip. (**c**) Photograph of the optical setup with microfluidic chip which was mounted between the aluminum brackets. (**d**) Speckle image of synthesized particles as obtained by the detection camera with indication of the measurement window.

The finished chip can be seen in Fig. 3a. To connect with the syringe pumps, the chip was placed between two mounting brackets made of aluminium with channel entries on the sides (see Fig. 3b). O-rings located between the holes in the aluminium and those in the glass substrate provided perfect sealing when pressed between the lower and upper brackets by screws. For the supply of liquids, the mounting bracket assembly was connected to the syringes with PTFE tubes and flangeless screws (Techlab GmbH, Braunschweig, Germany). Syringe pumps (Nemesys Base120 + low-pressure modules, Cetoni GmbH, Korbussen, Germany) equipped with glass syringes (2.5 mL glass syringe, Innovative Labour System, Stützerbach, Germany) were used to control liquid flow rates.

### Reagents

Throughout all precipitation experiments, 2.5 g L^− 1^ polysorbate 80 and 5 g L^− 1^ castor oil were added to absolute ethanol (*HPLC* grade solvent from Fisher Scientific, Loughborough, United Kingdom). The solutions were filtered with a 0.2 μm syringe filter (Puradisc 25 TF Whatman™, 0.2 μm polytetrafluoroethylene, GE Healthcare UK).

### Flow visualization

The flow conditions were experimentally examined by optical microscopy (Zeiss Axio Vert.A1, Zeiss, Oberkochen, Germany) using coloured solutions. 2 mg/mL methylene blue (Sigma Aldrich, Steinheim, Germany) dissolved in (Fisher Scientific, Loughborough, United Kingdom) representing the organic phase was injected through the nozzle, while deionized water was flushed through the main channel.

### Offline *DLS* measurement

Offline particle measurements were obtained with commercial *DLS* systems (Zetasizer Ultra, Malvern Panalytical GmbH and SZ-100, Horiba), which delivered consistent results.

### *FlowDLS* setup

As a light source, a relatively inexpensive diode laser (PLTB450B, Osram GmbH) emitting at a wavelength of 450 nm with 2000 mW optical output power was used. The collimated beam was directed through the back of the substrate of the *LARLM* chip onto the center of the microfluidic channel using an aspheric lens with a focal length of 7.5 mm. The focusing lens is kept at a distance further than its focal length to achieve a spot size of approx. 250 μm in the channel.

The microfluidic chip in mounting brackets was held in place on an optical table using a 3D-printed holder. In order to evaluate the *flowDLS* system, reference measurements were performed on commercial nanoparticles with narrow size distributions (Polybead Polystyrene Microspheres with a diameter of 50 nm from Polysciences, Inc. as well as *SiO*_*2*_*-F* silicon dioxide nanoparticles with diameters of 143 nm and 245 nm from microParticles GmbH). For some experiments, the chip was replaced by a square glass capillary. The light was collected at an initial angle of 90° using a 4× objective lens. The axes of the incident laser beam, flow direction, and detection direction were all aligned at right angles to each other (see Fig. 3c and Supporting Information 1). Using an additional objective lens and a CMOS camera the particle stream could be monitored for possible fouling or air introduced into the microfluidic system.

Unlike in conventional *DLS* systems, where detectors without a spatial resolution such as avalanche photodiodes or photomultiplier tubes are being used to detect the scattered light, we employed CMOS cameras (ace acA1440-220 μm, Basler AG) to capture a two-dimensional speckle image. Figure 3d shows a typical image of the speckles used for *flowDLS* (see also Supporting Information 2). By splitting the scattered light between two CMOS cameras, one triggered with a defined delay $\:\tau\:$ with respect to the other camera, allowed capturing correlation times much smaller than determined by the maximum frame rate of a single camera.

### *FlowDLS* algorithms

The degree of similarity between two images${\text{~}}{{\text{I}}_{\text{1}}}{\text{~}}$and${\text{~}}{{\text{I}}_{\text{2}}}$ can be compared using the cross-correlation function ${\text{\varvec{\upgamma}~(~}}{{\text{I}}_{\text{1}}}{\text{,~}}{{\text{I}}_{\text{2}}}{\text{~)}}$. In the following, the steps that were taken to determine the value of the autocorrelation function (*ACF*) $~{g_2}$ for a given time delay $\tau$ and to calculate the particle diameter are described. For eliminating influences of convection two different approaches (A) autocorrelation correction and (B) decay rate correction were followed.

(A) Autocorrelation correction.

Step 1: Compensation of slight misalignments between the two cameras. To align with camera image ${\text{~}}{{\text{I}}_{\text{1}}}{\text{~}}$ the image ${\text{~}}{{\text{I}}_{\text{2}}}$ required a transformation ${\text{I}}_{{{2}}}^{{{{\prime }}}}{{=}}{{\text{T}}_{{{\text{u}}_{{1}}}{{,}}{{\text{u}}_{{2}}}{{,}}\phi }}\left( {{{\text{I}}_{{2}}}} \right)$ involving a lateral shift by ${u_1}$ pixels in ${\text{x}}$ direction and ${\text{~}}{{\text{u}}_{\text{2}}}{\text{~}}$ pixels in ${\text{y}}$ direction, as well as a rotation by an angle $\phi$. The parameters of this transformation were determined by taking two images with the camera at the same time (${\text{\varvec{\uptau}=0}}$) and numerically finding ${{\text{u}}_{\text{1}}}$, ${{\text{u}}_{\text{2}}}$ and $\phi$, for which a maximum was found in the cross correlation $\gamma$:

1 $${\text{\varvec{\upgamma}}}\left( {{{\text{I}}_{\text{1}}}{\text{,I}}_{{\text{2}}}^{{{\prime }}}} \right){\text{=}}\frac{{\mathop \sum \nolimits_{{{\text{x,y}}}} \left[ {{{\text{I}}_{\text{1}}}\left( {{\text{x,y}}} \right){\text{-}}\overline {{{{\text{I}}_{\text{1}}}_{{{\text{x,y}}}}}} } \right]\left[ {{\text{I}}_{{\text{2}}}^{{{\prime }}}\left( {{\text{x,y}}} \right){\text{-}}\overline {{{\text{I}}{{_{{\text{2}}}^{{{\prime }}}}_{{\text{x,y}}}}}} } \right]}}{{{{\left\{ {\mathop \sum \nolimits_{{{\text{x,y}}}} \left[ {{{\text{I}}_{\text{1}}}\left( {{\text{x,y}}} \right){\text{-}}\overline {{{{\text{I}}_{\text{1}}}_{{{\text{x,y}}}}}} } \right]} \right.}^{\text{2}}}\mathop \sum \nolimits_{{{\text{x,y}}}} {{\left. {{{\left[ {{\text{I}}_{{\text{2}}}^{{{\prime }}}\left( {{\text{x,y}}} \right){\text{-}}\overline {{{\text{I}}{{_{{\text{2}}}^{{{\prime }}}}_{{\text{x,y}}}}}} } \right]}^{\text{2}}}} \right\}}^{{\text{0,5}}}}}}$$

Here, ${{\text{\varvec{\Sigma}}}_{{\text{x,y}}}}$ denotes the summation over an image section chosen small enough to be in the overlapping area of the two images even after shifting and rotating image ${\text{~}}{{\text{I}}_{\text{2}}}$.

Step 2: Determination of the flow direction. A delay of $\tau >0$, typically $100$ µs, was chosen between triggering the capture of the two images ${I_1}\left( {{\text{t=0}}} \right)$ and ${{\text{I}}_{\text{2}}}\left( {{\text{t=\varvec{\uptau}}}} \right)$ with particles under flow. In addition to the static transformation in step 1, a shift leading to ${\text{I}}_{2}^{{\prime\prime}} (\tau )$ was then required to align the two images. This shift in the x, y-plane denoted by the components ${u_{{\text{x}},{\text{flow}}}}$ and ${u_{{\text{y}},{\text{flow}}}}$ was found by maximizing the cross correlation $\gamma ({\text{I}}_{1} (0),{\text{I}}_{2}^{\prime\prime} (\tau ))$. The flow direction ${\vec {e}_{{\text{flow}}}}$ was determined as a vector of unit length from ${{\text{u}}_{{\text{x,~flow}}}}$ and ${{\text{u}}_{{\text{y,~flow}}}}$.

Step 3: Obtaining a corrected ACF. The laser and cameras were triggered at a series of different delays $${\tau _i}$$ to obtain a stream of image pairs $${{\text{I}}_{\text{1}}}\left( {\text{0}} \right){\text{,}}$$$${{\text{I}}_{\text{2}}}\left( {{{\text{\varvec{\uptau}}}_{{\text{i~}}}}} \right)$$. To every image $${{\text{I}}_{\text{2}}}\left( {{{\text{\varvec{\uptau}}}_{\text{i}}}} \right){\text{,}}$$ the static transformation $${{\text{T}}_{{{\text{u}}_{\text{1}}}{\text{,}}{{\text{u}}_{\text{2}}}{\text{,}}\phi }}$$ was applied resulting in $${I_1}\left( 0 \right){\text{~}}$$and $${\text{I}}_{{\text{2}}}^{{{\prime }}}\left( {{{\text{\varvec{\uptau}}}_{\text{i}}}} \right)$$. Uncorrected ACF $${{\text{g}}_{\text{2}}}\left( \tau \right)=~~{\text{\varvec{\upgamma}}}\left( {{I_1}\left( 0 \right),I_{2}^{{\prime }}\left( {{\tau _i}} \right)} \right)$$ was obtained with the image pairs $${{\text{I}}_{\text{1}}}\left( {\text{0}} \right){\text{~}}$$and $${\text{I}}_{{\text{2}}}^{{{\prime }}}\left( {{{\text{\varvec{\uptau}}}_{\text{i}}}} \right)$$. For the autocorrelation correction, a scalar shift $$s\left( {{\tau _i}} \right)$$ in direction of $${\vec {e}_{{\text{flow}}}}$$ was determined by finding the maximum of $${{\text{g}}_{\text{2}}}\left( {\text{\varvec{\uptau}}} \right)$$ for every image pair. A Nelder-Mead algorithm was used for the maximum finding, where the computational effort could drastically be reduced to a one-dimensional problem by knowing the flow direction $${\vec {e}_{{\text{flow}}}}$$ from step 2. An example of real time acquisition of autocorrelation and image shift is given in Supporting Information 3.

With determined shifts s(τi), the speed v of the particles was determined as

2 $${\text{v=}}\frac{{\mathop \sum \nolimits_{{\text{i}}} {\text{s}}\left( {{{\text{\varvec{\uptau}}}_{\text{i}}}} \right)}}{{\mathop \sum \nolimits_{{\text{i}}} {{\text{\varvec{\uptau}}}_{\text{i}}}}}{\text{.}}$$

Particles that are subject to Brownian motion exhibit a decreasing correlation over time. Thus, the algorithm to maximize the correlation between the image pairs failed to find a sensible shift ${\text{s}}\left( {{{\text{\varvec{\uptau}}}_{\text{i}}}} \right)$ for larger ${\text{\varvec{\uptau}}}$. A cut-off condition was therefore introduced into the summation of Eq. 2. Any $s\left( {{\tau _i}} \right)$ and ${\tau _i}$ summands that satisfied the condition ${{\text{g}}_{\text{2}}}\left( {{{\text{\varvec{\uptau}}}_{\text{i}}}} \right){\text{<0}}{\text{0.3~}}$were not considered.

Step 4: Determining the particle diameter and PDI. A least-squares fit of the shift corrected ${{\text{g}}_{\text{2}}}\left( {\text{\varvec{\uptau}}} \right)$ was performed with an exponential function as described in the literature^54^.

3 $${g_2}\left( \tau \right){\text{=B+\varvec{\upbeta}exp}}\left( {{\text{-2\varvec{\Gamma}\varvec{\uptau}}}} \right) \cdot {\left( {{\text{1+}}\frac{{{{\text{\varvec{\upmu}}}_{\text{2}}}}}{{{\text{2!}}}}{\text{+ \ldots }}} \right)^{\text{2}}}$$

Here ${\text{B}}$ is the baseline, ${\text{\varvec{\upbeta}}}$ the instrument response, ${\text{\varvec{\Gamma}}}$ the mean decay rate, and ${{\text{\varvec{\upmu}}}_{\text{2}}}$ is the second moment about the mean of the decay rate distribution. The decay rate ${{\varvec{\Gamma}}}$ can be related to the diffusion coefficient D as

4 $${\text{D=}}\frac{{\text{\varvec{\Gamma}}}}{{{{\text{q}}^{\text{2}}}}}{\text{,}}$$

where ${\text{q=}}\frac{{{\text{4\varvec{\uppi}}}{{\text{n}}_{\text{0}}}}}{{\text{\varvec{\uplambda}}}} \cdot {\text{sin}}\left( {\frac{{\text{\varvec{\uptheta}}}}{{\text{2}}}} \right)$ is the scattering vector, with ${{\text{n}}_{\text{0}}}{\text{=1}}{\text{0.33}}$ the refractive index of the solvent, ${{\varvec{\uplambda}=450}}$ nm the wavelength of the laser, and ${{\varvec{\uptheta}=90^\circ }}$ the scattering angle.

5 $${{\text{d}}_{\text{H}}}{\text{=}}\frac{{{{\text{k}}_{\text{B}}}{\text{T}}}}{{{\text{3\varvec{\uppi}\varvec{\upeta}}} \cdot {\text{D}}}}{\text{,}}$$

The particle hydrodynamic diameter${\text{(}}{{\text{d}}_{\text{H}}}{\text{)~}}$ can then be determined using the Einstein-Stokes relation

where ${{\text{k}}_{\text{B}}}$ is the Boltzmann constant, ${\text{T}}$ is the temperature, and ${\text{\varvec{\upeta}}}$ the dynamic viscosity of the solvent taken from literature. The PDI is given by

6 $${\text{PDI=}}\frac{{{\text{2}}{{\text{\varvec{\upmu}}}_{\text{2}}}}}{{{{\text{\varvec{\Gamma}}}^{\text{2}}}}}{\text{.}}$$

**Fig. 4:**
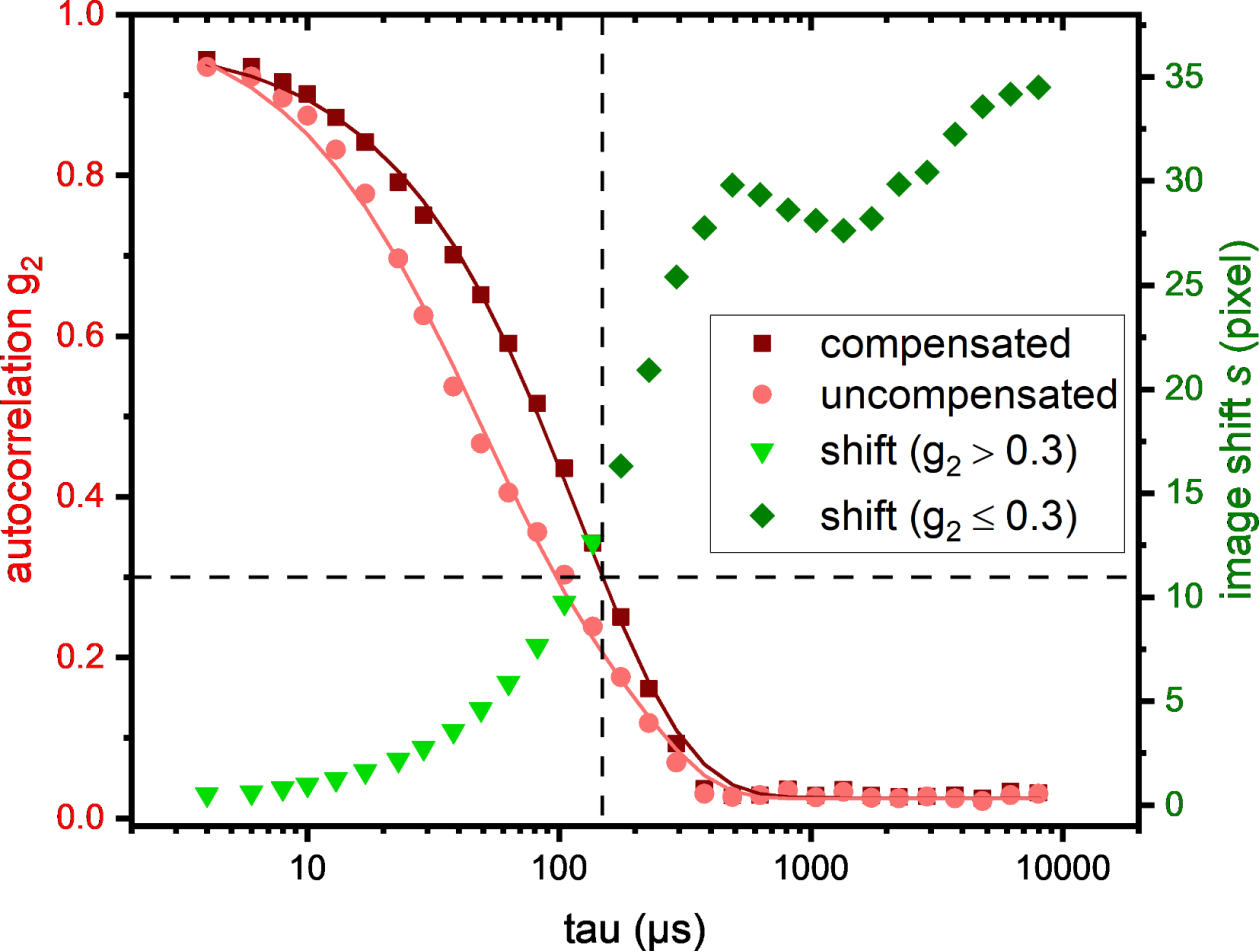
Autocorrelation ${g_2}\left( {\text{\varvec{\uptau}}} \right)$ of an exemplary measurement with and without autocorrelation correction. The curves represent least square fits assuming a function as given in Eq. (3). The cut-off condition is depicted by dashed lines. In addition, the shifts *s(τ)* determined for the correction are given.

Figure 4 illustrates that the corrected ${g_2}\left( \tau \right)$ decays faster than the uncorrected ${g_2}\left( {\text{\varvec{\uptau}}} \right)$ and prevents a considerable underestimation of the particle radius. In the given example, the radius determined with correction was 80.6 nm compared to a value of 37.1 nm without correction. Additionally, the shape of the uncorrected correlation function deviates from a simple exponential decay, which led to a vastly overestimated PDI of 0.2 versus a PDI of 0.1 in the corrected case.

(B) Decay rate correction.

Steps 1 and 2 were followed in the same manner as before. In step 3, only the uncorrected ${{\text{g}}_{\text{2}}}\left( {\text{\varvec{\uptau}}} \right)$ was determined. As step 4 a least-squares fit of the uncorrected ${{\text{g}}_{\text{2}}}\left( {\text{\varvec{\uptau}}} \right)$ was performed for varied flow velocities to determine decay rates ${{\text{\varvec{\Gamma}}}_{\text{v}}}$. A rate correction was used assuming a linear relation

7 $${{\text{\varvec{\Gamma}}}_{\text{0}}}{\text{=\varvec{\Gamma}(v)-m}} \cdot {\text{v}}{\text{.}}$$

With ${\text{m}}$ obtained through a linear fit, a corrected decay rate ${{\text{\varvec{\Gamma}}}_0}$ could be calculated for any velocity and inserted in Eq. (4) to determine a decay rate corrected hydrodynamic diameter on the basis of Eq. (5).

## Results and discussion

### Organic solvent injection and distribution in the *LARLM*

**Fig. 5:**
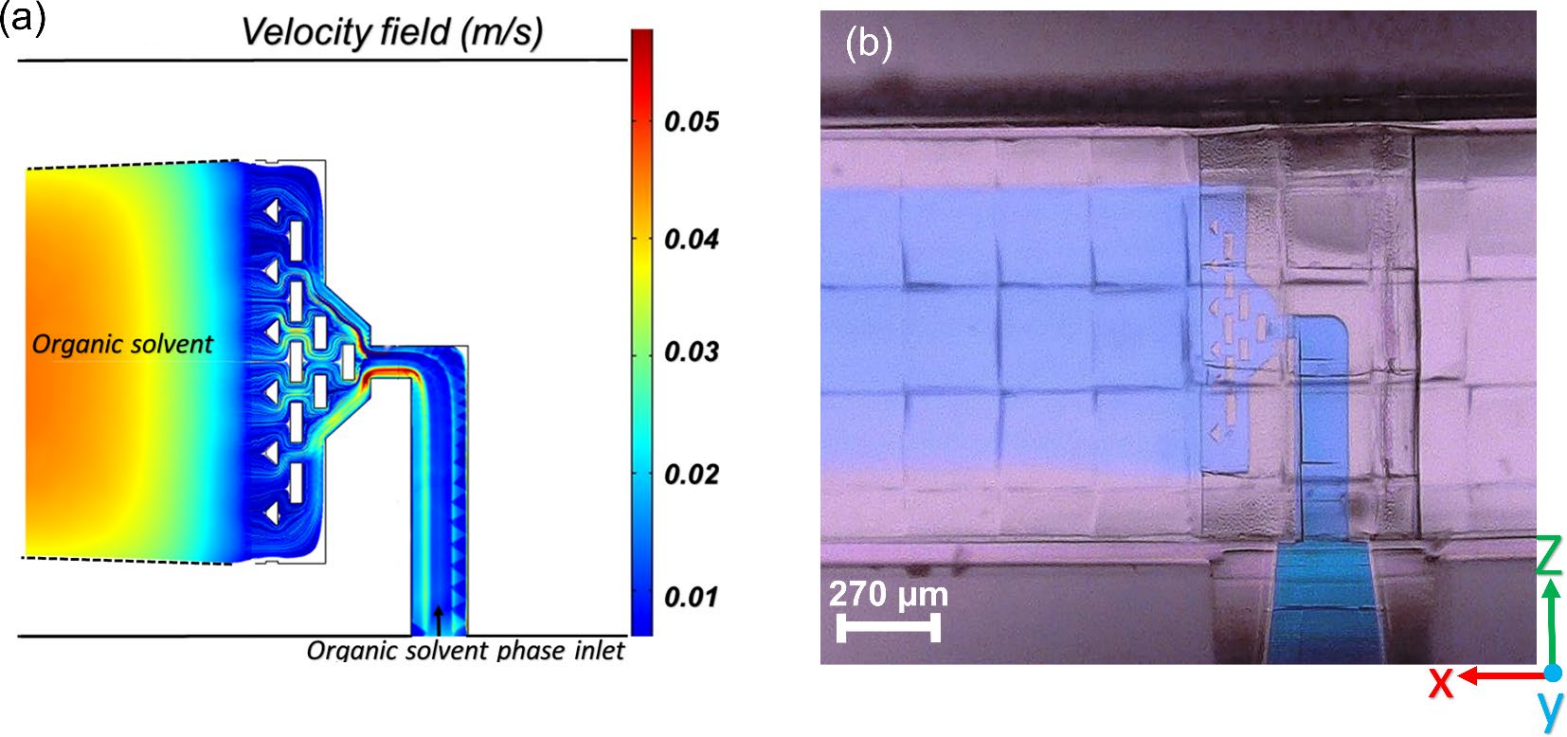
(**a**) Flow stream simulation of the injection at $\:{Q}_{aqueous}$= 90 µl/min for the aqueous phase and $\:{\text{Q}}_{\text{organic}}$ = 10 µl/min. The velocities range between 0.01 m/s (blue) and 0.05 m/s (red). (**b**) Microscopic image of the injected phase coloured with methylene obtained with identical volume flow rates.

*CFD* simulations were applied to study the flow in the *LARLM* system while neglecting diffusion. The simulated injection stream as displayed in Fig. 5a, proved that the organic phase leaves the nozzle with an almost perfectly homogenized velocity and then forms a thin layer that stays surrounded by the aqueous phase. The boundaries between two fluids depended on $\:{\text{Q}}_{\text{aqueous}}\text{/}{\text{Q}}_{\text{organic}}$. The experiment with coloured injection fluid (see Fig. 5b) was consistent with the simulation.

To achieve a consistent supersaturation environment in the LARLM system, it was essential to rapidly mix the organic phase with the aqueous phase. This mixing is facilitated by controlled diffusion through a very thin layer, resulting in a high density of nucleations. With more nucleation, particles have limited growth, leading to the production of smaller and more monodisperse nanoparticles. As in our previous works on microfluidic precipitation of castor oil nanoparticles^12,45^ the efficiency of diffusion was enhanced by the increased contact between the thin sheet of organic phase and surrounding aqueous phases which could be controlled in the LARLM. At the considered flow rate ratios inside the LARLM system, the organic phase was fully wrapped by the aqueous solution and fouling, which is one of the biggest challenges in microfluidic nanoparticle precipitation, could not happen.

**Fig. 6:**
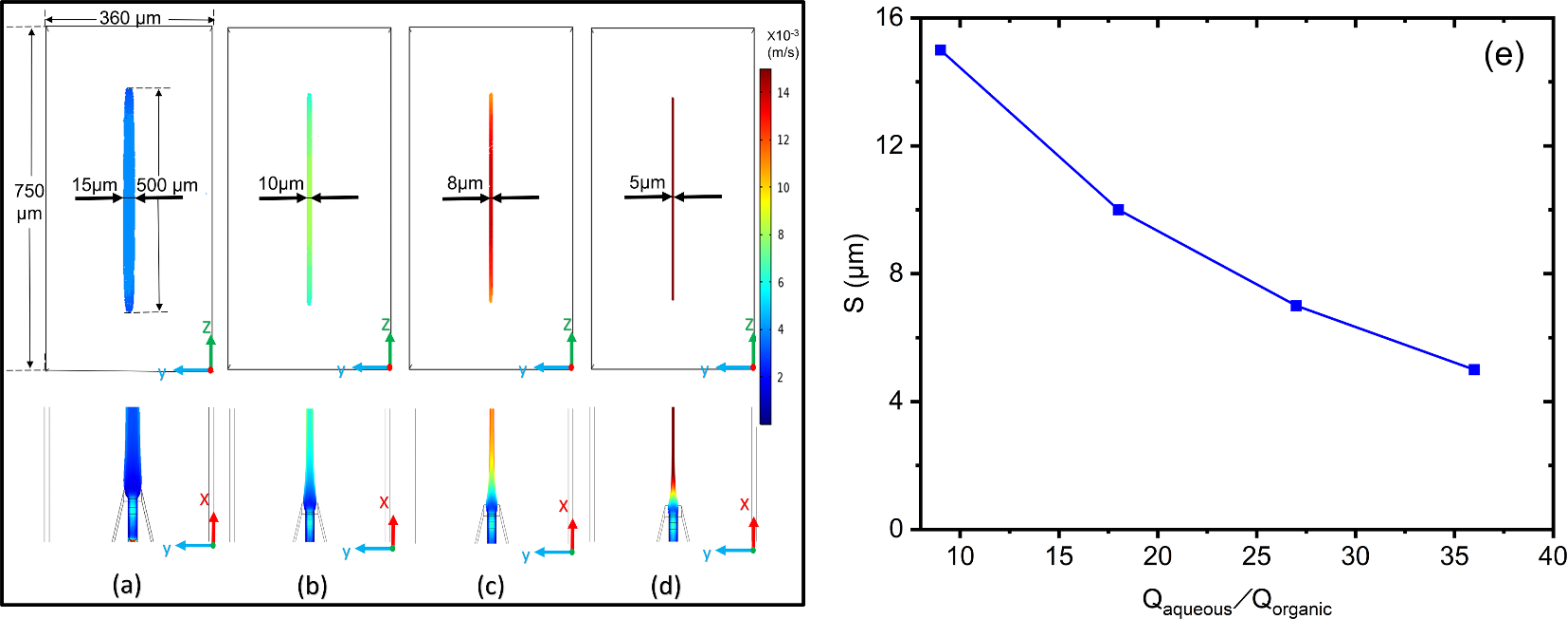
Cross sections in flow direction (lower row) and perpendicular to it at about 6.5 mm after the nozzle (upper row) showing the thickness S of the organic phase sheet and colour coded velocities of the organic phase as obtained by CFD simulations for volume flow rate ratios $\:{\text{Q}}_{\text{aqueous}}\text{/}{\text{Q}}_{\text{organic}}$ of (**a**) 9, (**b**) 18, (**c**) 27, (**d**) 36. (**e**) The thickness S of thin sheet of solvent phase as fonction of $\:{\text{Q}}_{\text{aqueous}}\text{/}{\text{Q}}_{\text{organic}}.$

Figure 6a-d shows the cross-sectional shapes of the thin sheet of the organic phase after injection as obtained for different flow rate ratios. The solvent sheet thickness $\:\text{S}$ continuously reduces with increasing $\:{\text{Q}}_{\text{aqueous}}\text{/}{\text{Q}}_{\text{organic}}$. Figure 6e shows the dependence of $\:\text{S}$ on $\:{\text{Q}}_{\text{aqueous}}\text{/}{\text{Q}}_{\text{organic}}$. This allows to precisely control the speed of mixing since the time required for complete mixing by diffusion $\:\text{\:}{\text{t}}_{\text{mix}}$ is proportional to $\:{\text{S}}^{\text{2}}$. For smaller $\:\:{\text{t}}_{\text{mix}}$ the nucleation occurs faster. Consequently, smaller nanoparticles are expected to be generated. Due to the intensified diffusion through and within the thin sheet, many nuclei will be formed in the initially highly supersaturated zone, but their growth is limited by the rapidly decreasing supersaturation. It can be expected that the LNP will become smaller as the ratio $\:{\text{Q}}_{\text{aqueous}}\text{/}{\text{Q}}_{\text{organic}}$ is reduced but not with any change in the total flow rate $\:{\text{Q}}_{\text{aqueous}}\text{+}{\text{Q}}_{\text{organic}}$ However, the latter can compensate for the loss of nanoparticle productivity associated with smaller $\:{\text{Q}}_{\text{aqueous}}\text{/}{\text{Q}}_{\text{organic}}$ Moreover, because this thin sheet was positioned in the center of the microchannel around the maximum of the parabolic flow profile the velocity gradients were not only small in $\:z$ direction because of nearly one-dimensional flow but also negligible in $\:\text{y}$ direction. In contrast to the situation in a typical glass capillary without nozzle injection, this promised very low variations in the velocities of precipitated nanoparticles and better applicability of the autocorrelation correction for *flowDLS* as described in the previous section.

### Nanoparticle precipitation in the *LARLM*

**Fig. 7:**
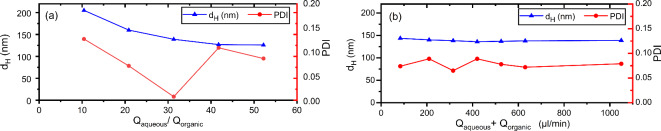
(**a**) Hydrodynamic diameters $\:{d}_{H}$ and PDI as obtained by offline *DLS* for varied $\:{\text{Q}}_{\text{aqueous}}\text{/}{\text{Q}}_{\text{organic}}.$ at constant $\:{Q}_{aqueous}=20\:$µl/min. (**b**) $\:{d}_{H}$ and *PDI* as obtained with offline *DLS* for varied values of $\:{\text{Q}}_{\text{aqueous}}\text{+}{\text{Q}}_{\text{organic}}$at constant $\:{\text{Q}}_{\text{aqueous}}\text{/}{\text{Q}}_{\text{organic}}$ of 20.

Nanoparticles produced in the *LARLM* system were in a first step collected at the outlet and measured with offline *DLS*. In one experiment$\:{\text{Q}}_{\text{aqueous}}$was increased stepwise from 90 µl/min to 450 µl/min, while $\:{\text{Q}}_{\text{organic}}$was held constant at 20 µl/min. Figure 7a illustrates that nanoparticle size was monotonously reduced with increasing $\:{\text{Q}}_{\text{aqueous}}\text{/}{\text{Q}}_{\text{organic}}\:$but polydispersity did not follow a monotonous decay. While $\:{\text{Q}}_{\text{aqueous}}\text{/}{\text{Q}}_{\text{organic}}$ changed from 10 to 60, particle sizes decreased from 210 nm to 130 nm, but the *PDI* fluctuated between 0.01 and 0.12, indicating very monodisperse nanoparticles.

In the next experiment, $\:{\text{Q}}_{\text{aqueous}}\text{/}{\text{Q}}_{\text{organic}}\:$remained constant at 20, while $\:{\text{Q}}_{\text{aqueous}}\text{+}{\text{Q}}_{\text{organic}}$ increased from 100 µl/min to 1,000 µl/min. As expected, Fig. 7b shows that increasing $\:{\text{Q}}_{\text{aqueous}}\text{+}{\text{Q}}_{\text{organic}}$ did not have a significant effect on the nanoparticle sizes while the *PDI* fluctuated around 0.08.

### *FlowDLS* measurements in a glass capillary

**Fig. 8:**
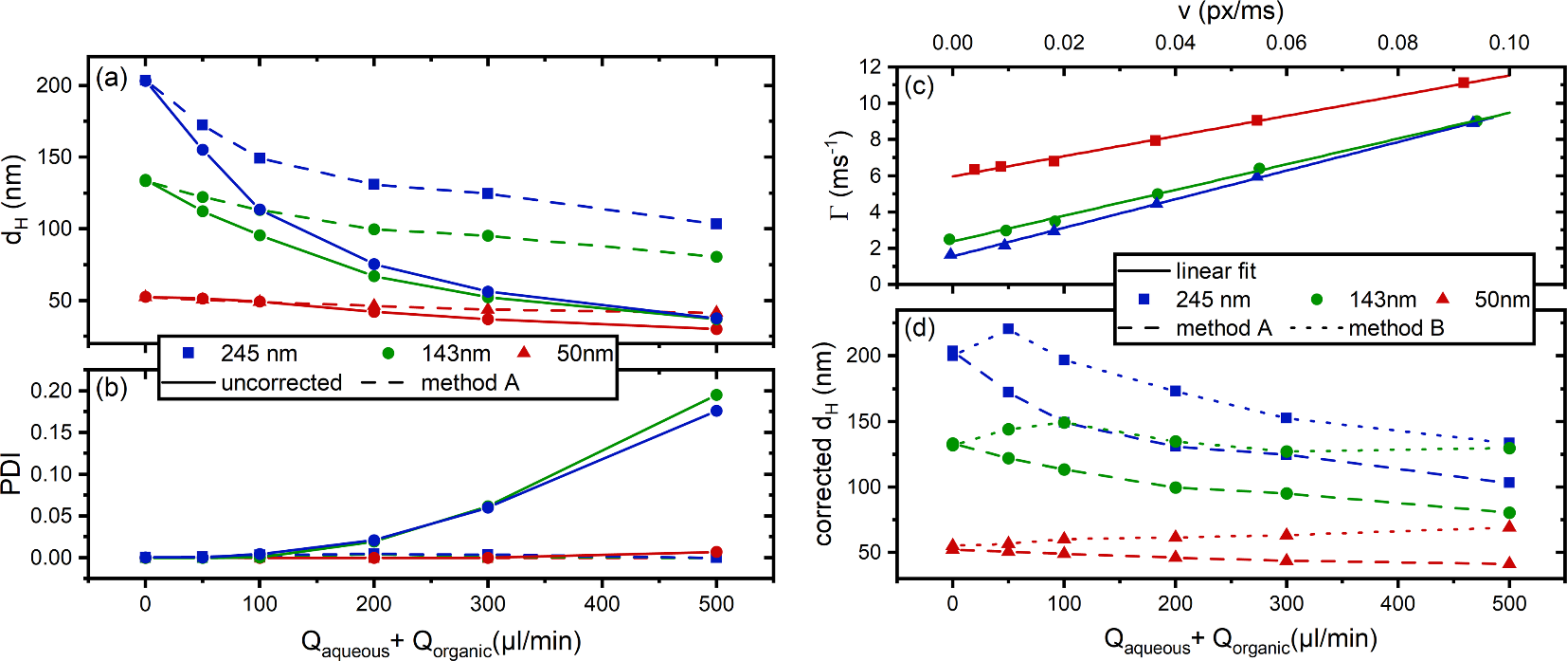
(**a**) $\:{d}_{H}$ obtained by *flowDLS* for commercial reference nanoparticles flowing through a glass capillary with and without correction method A. (**b**) *PDI* values obtained with and without correction method A. (**c**) Uncorrected particle decay rate versus measured velocity, showing good linearity. (**d**) Particle sizes corrected using either method A or B, obtained for varied volume flow rates.

As a first test of the correction algorithms of the *flowDLS*, suspensions with commercial model nanoparticles with diameters of 50 nm, 143 nm and 245 nm with very narrow size distributions ($\:PDI\approx\:0)\:$were pumped through a glass capillary with a quadratic inner cross-sectional area of 500 μm × 500 μm with flow rates of up to 500 µl/min (corresponding to an average velocity of 33 mm/s). In Fig. 8, particle size results obtained with autocorrelation correction (method A) and without are displayed. Figure 8a shows that without correction, diameters were heavily underestimated at flow rates of 200 µl/min and above, and larger particles with diameters of 143 nm and 245 nm could not even be discriminated. Figure 8b shows that with autocorrelation correction, the obtained *PDI* values remained close to zero across all tested flow rates, whereas without correction the *PDI* values increased for larger particles at flow rates above 200 µl/min. Even though the autocorrelation correction led to vastly improved results, the influence of the flow was not, in all cases, eliminated. For the laminar flow in the glass capillary with a parabolic velocity distribution profile, the autocorrelation correction assuming homogeneous velocity is too inaccurate, thus leading to an underestimation of the particle radius. A deeper discussion of these effects can be found in the literature^56,57^.

Therefore, we also applied the decay rate correction (correction method B) to our dat. Figure 8c shows the decay rates of the uncorrected $\:{\text{g}}_{\text{2}}\left(\tau\:\right)$ versus the velocity *v* as determined in step 3 for each particle size with linear fits according to Eq. 7. The values of $\:m$ were taken to correct the decay rates and to calculate the decay rate corrected *d*_*H*_ as shown in Fig. 8d across the investigated flow rate range.

Since larger particles are affected more by shear resulting from parabolic flow velocity profiles than smaller ones, the slopes needed for correction are larger (see Fig. 8c). This leads to an overestimation of the particles’ sizes for smaller particles and an underestimation for larger ones. For high flow rates, the measured decay rates start to overlap, and thus also, method B fails to properly distinguish between particle sizes. The *flowDLS* measurement in the glass capillary clearly indicates that flow velocity inhomogeneities have to be reduced to allow corrections that can compensate the convective movement of particles.

### In-situ *flowDLS* measurement in the *LARLM*

**Fig. 9:**
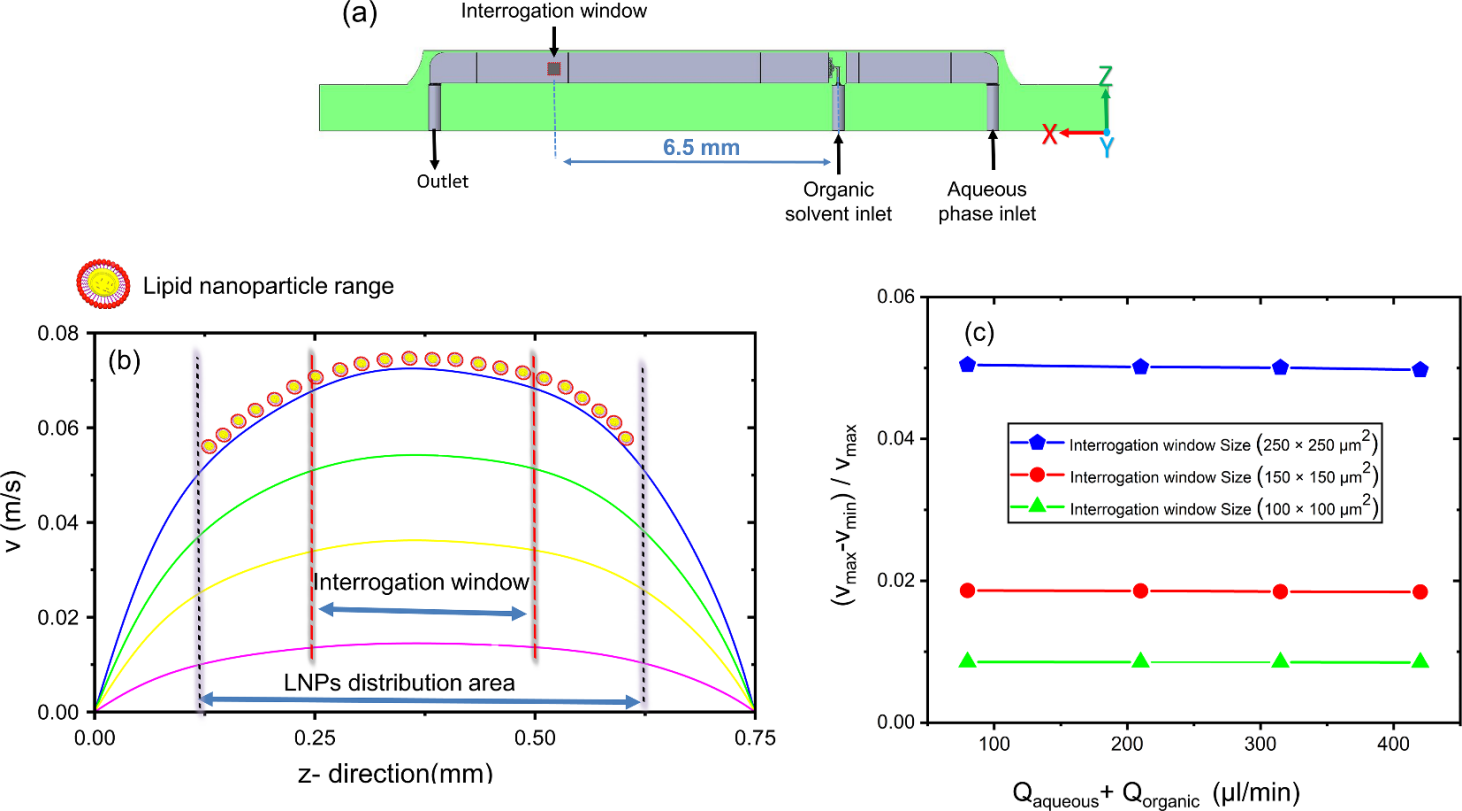
(**a**) True to scale schematic cross section illustrating area and position of the interrogation window within the microchannel. (**b**) Simulated flow velocity v in x direction obtained in the center of the channel in Y direction for different values of $\:{\text{Q}}_{\text{aqueous}}\text{+}{\text{Q}}_{\text{organic}}\:$ purple : 84 (µl/min), yellow : 210 (µl/min), green : 315 (µl/min), blue : 420 (µl/min)) at a constant value of $\:{\text{Q}}_{\text{aqueous}}\text{/}{\text{Q}}_{\text{organic}}\text{.}=20$. (**c**) Relative velocity variation $\:\left({\text{v}}_{\text{max}}\text{-}{\text{v}}_{\text{min}}\right)\text{/}{\text{v}}_{\text{max}}\text{\:}$ decreased with reducing the size of the interrogation window from 250 × 250 µm^2^ (blue) to 150 × 150 µm^2^ (yellow) to 100 × 100 µm^2^ (purple).

At about 6.5 mm behind the organic solvent nozzle, the measurement window was set (see Fig. 9a). An analytical estimation of the diffusion of the ethanol stream in water (using $\:\text{Δx}\text{\:}\text{=}\sqrt{\text{2Dt}}$ ) shows that for an initial thickness of the ethanol sheet of 20 μm, the interdiffusion is complete after about *t* = 50 ms. This is a much shorter timescale than the particles need to reach the measurement area even at the highest flow rate used in the experiments (125 ms for the total flow rate of 420 µl/min). In other words, the precipitation was certainly completed when the measurement window was reached.

At $\:{\text{Q}}_{\text{aqueous}}\text{/}{\text{Q}}_{\text{organic}}$ of 20, the solution stream and therefore the distribution of precipitated nanoparticles covered a range of 0.5 mm in $\:z$ direction. The simulations revealed that the thin sheet carrying the particles still experiences some small velocity variation in $\:z$ direction (see Fig. 9a).

Nanoparticles closer to the wall had a lower velocity than those in the channel center. Since this can lead to inaccuracies in the autocorrelation correction, it was necessary to limit the optical interrogation window of the *flowDLS* to further lower the relative velocity differences $\:\frac{\left({\text{v}}_{\text{max}}\text{\:\:-}{\text{v}}_{\text{min}}\right)}{{\text{v}}_{\text{max\:}}}$. However, with a too small interrogation window, the noise would increase. As a compromise between velocity homogeneity and measurement speed, we chose a window of 100 × 100 µm^*2*^ corresponding to 100 × 100 px^*2*^ (see Figs. 3b and 9b). In this window (see Fig. 9c) the velocity variation became quite small and acceptable values of $\:\frac{\left({\text{v}}_{\text{max}}\text{-}{\text{v}}_{\text{min}}\right)}{{\text{v}}_{\text{max\:}}}$< 0.01 were reached at all considered total volume flow rates.

**Fig. 10:**
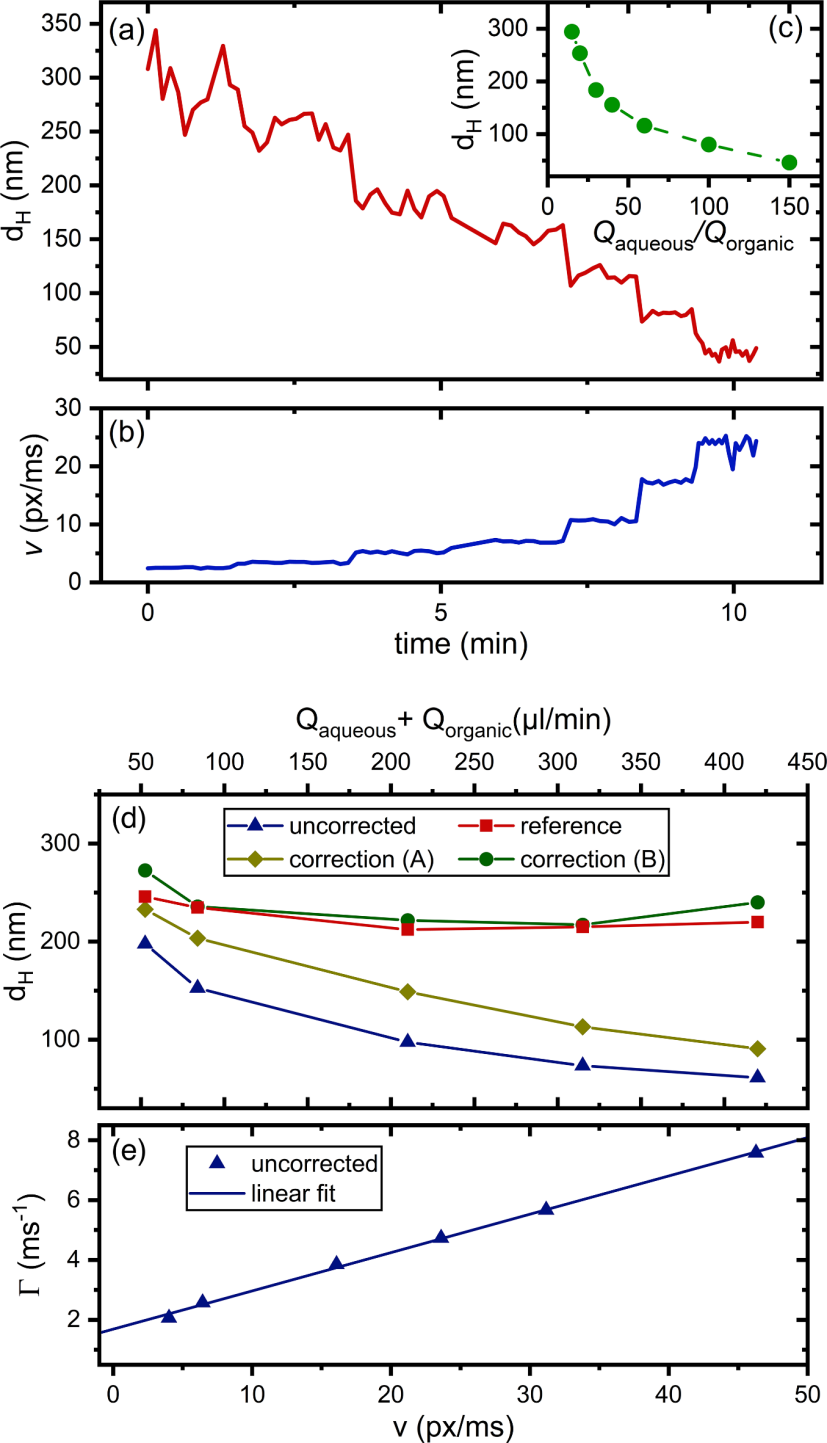
(**a**) Nanoparticle diameter obtained with correction method A and (**b**) velocities as obtained by in-situ *flowDLS* at ${\text{Q}}_{{{\text{Organic}}}} = 2.5\;\upmu {\text{l}}/\min$ and varied $\:{\text{Q}}_{\text{aqueous}}\text{/}{\text{Q}}_{\text{organic}}$ (**c**) dependence of determined nanoparticle diameter on $\:{\text{Q}}_{\text{aqueous}}\text{/}{\text{Q}}_{\text{organic}}$ (**d**) comparison of *flowDLS* measurements with a reference offline measurement. The flow rate ratio $\:{\text{Q}}_{\text{aqueous}}\text{/}{\text{Q}}_{\text{organic}}$ was kept constant at 20 while varying the total flow rate. The data was generated without any correction, as well as with autocorrelation correction (method A) and decay rate correction (method B). (**e**) For the decay rate correction (method B), the uncorrected decay rate $\:\text{Γ}$ is plotted vs. the measured velocity $\:\text{v}$ of the particles, which were synthesized at 210 µl/min. A linear fit was applied to the measurements.

To investigate the performance of the *flowDLS in-situ* measurements in the *LARLM*, $\:{Q}_{aqueous}$ was varied in an experiment in steps from 37.5 µl/min to 375 µl/min while keeping $\:{Q}_{organic}$ constant at 2.5 µl/min. The autocorrelation function was determined every 7.6 s from $\:\tau\:=0\:\text{t}\text{o}\:2\:\text{m}\text{s}\:$in 51 logarithmically distributed steps. Figure 10 shows the *flowDLS* particle diameter obtained with autocorrelation correction but without decay rate correction versus time during the precipitation experiment. With every stepwise increase of $\:{\text{Q}}_{\text{aqueous}}\text{/}{\text{Q}}_{\text{organic}}.$ in the *LARLM*, a step in both the diameter (Fig. 10a) and velocity (Fig. 10b) was discernible. Figure 10c shows the nanoparticle diameters averaged within each interval of constant flow condition as a function of $\:{\text{Q}}_{\text{aqueous}}\text{/}{\text{Q}}_{\text{organic}}$. Such dependencies could be determined with our device within minutes, which is impossible using offline *DLS*.

In the next experiment, the flow rate ratio $\:{\text{Q}}_{\text{aqueous}}\text{/}{\text{Q}}_{\text{organic}}.$ was kept constant at 20 while the total flow of $\:{Q}_{aqueous}+{Q}_{organic}$was varied between 52.5 and 420 µl/min. At each total flow rate, the corrected *ACF* as well as the uncorrected *ACF* was determined from in-situ obtained f*lowDLS* speckle images. The suspensions with precipitated nanoparticles obtained for each flow rate were collected and measured also offline using a commercial *DLS* instrument. In accordance with the previous experiment (Fig. 7a, b), the particle sizes measured offline were practically not varying with the total flow rate. When using no correction or when applying the autocorrelation correction (method A), the in-situ determined diameters by the *flowDLS* tended to be smaller than the diameters determined offline. Similar to the measurements of model particles in the glass capillary (see Fig. 8), the uncorrected diameters were smaller than the corrected ones using method A. This could result from the strong dependency of dynamic viscosity from the ratio of ethanol to water in the mixture^57^. With a not precisely known dynamic viscosity for the mixture, the particle diameter determined by the *flowDLS* can be underestimated (see Eq. 5). This could also be an explanation for an incomplete mixture of the solvent over the full microchannel cross section at the region of *in-situ DLS* measurement. To apply the decay rate correction without the possible influence of viscosity we analyzed the influence of flow rates using particles, which were synthesized at a total flow rate of 210 µl/min through the nozzle. As a liquid for the main channel flow, we chose a mixture of 4.8% ethanol in water, which corresponds to the ethanol content in the particle solution synthesized at a flow rate ratio of $\:{Q}_{\text{a}\text{q}\text{u}\text{e}\text{o}\text{u}\text{s}}/{Q}_{\text{o}\text{r}\text{g}\text{a}\text{n}\text{i}\text{c}}=20$.

By measuring the uncorrected decay rates and particle velocities at varying total flow rates but at a constant flow rate ratio of outer to inner flow of 20:1 (see Fig. 10e), we were able to determine the calibration constant $\:m$ describing the increase in decay rate with increasing flow. The result of applying this decay rate correction (method B) according to Eq. 7 is also shown in Fig. 10d. The comparison to the reference offline measurement suggests that applying a decay rate correction (method B) leads to the most accurate result, whereas applying autocorrelation correction (method A) can lead to an underestimation of particle sizes.

The successful suppression of influence of particle velocities with method B, which was not possible in the capillary measurement, became possible in the *LARM*, because the particles were concentrated in the center of the channel, so that they exhibited very similar velocities, whereas in the capillary, the particles are spread and subjected to larger shear.

## Conclusion and outlook

The unique *LARLM* device allows for a homogeneous injection of the organic phase into the aqueous phase in the form of a thin sheet, thus increasing the contact area. Three major advantages are thereby combined: (1) the channel wall is never exposed to the organic phase and no fouling occurs, (2) the intermixing of solvent and antisolvent by diffusion becomes extremely fast and (3) the thin sheet carrying the precipitated nanoparticles is centered around the maximum of the parabolic flow profile which homogenizes the velocity of nanoparticles. While (1) and (2) allow for perfected nanoparticle precipitation, (3) in combination with the optical accessibility of the micro channel enabled *flowDLS* for precipitated particles. With a suitable correction algorithm, the determined particle sizes became consistent with offline measurement. The necessary correction of decay rates only requires that a calibration is carried out in advance with any sort of nanoparticles whose sizes may even be unknown. By integrating the *flowDLS* with the microfluidic synthesis chip, we were able to measure particle sizes directly after their synthesis. This is the first time there that is a possibility for dynamic feedback control and long-term stabilized nanoparticle precipitation.

In the future, our technology could be used to monitor nanoparticles within only a few milliseconds after their nucleation. The position of the measurement volume or the flow rate could act as the representative of the time axis for their formation process. Together with other tools such as molecular dynamics simulations, completely novel insights into particle-forming processes might be gathered in this manner. For pharmaceutical companies, this enables a pathway towards speedy process developments at minimal sample volumes, which is desperately needed for processes such as lipid nanoparticle formulation involving costly chemicals.

## Electronic supplementary material

Below is the link to the electronic supplementary material.

Supplementary Material 1

Supplementary Material 2

Supplementary Material 3

Supplementary Material 4

## Data Availability

The datasets used and/or analyzed during the current study are available from the corresponding author upon reasonable request.
