# Supplementary material for: Diffusive micromixing combined with dynamic in situ laser scattering allows shedding light on lipid nanoparticle precipitation
Source: Sci Rep. 2024 Oct 17;14:24356. doi: 10.1038/s41598-024-73721-0 (PMC11487189; doi:10.1038/s41598-024-73721-0)
Supplement: Supplementary file 4 — Supplementary Material 4 [file 41598_2024_73721_MOESM4_ESM.docx]

**Supplementary material:**

**Optical setup with microfluidic chip** (supporting Information 1)

The video shows the LARLM chip within the *FlowDLS* setup during measurement

**Speckle image** (supporting Information 2)

The video shows the speckle formation origoination from particles precipitateted in the LARL

**Autocorrelation function** (supporting Information 3)

The video shows the real time acquisition of autocorrelation and image shift during measurement.
